# HIV-I Nef inhibitors: a novel class of HIV-specific immune adjuvants in support of a cure

**DOI:** 10.1186/s12981-017-0175-6

**Published:** 2017-09-12

**Authors:** Gregory A. Dekaban, Jimmy D. Dikeakos

**Affiliations:** 1 Department of Microbiology and Immunology, Schulich School of Medicine and Dentistry, The University of Western Ontario, Dental Sciences Building, Room 3007J, London, ON N6A 5C1 Canada; 2 BioTherapeutics Research Laboratory, Molecular Medicine Research Laboratories, Robarts Research Institute, The University of Western Ontario, Room 2421A, London, ON N6A 5B7 Canada

**Keywords:** HIV-1, Nef, Latency, Vaccines

## Abstract

The success of many current vaccines relies on a formulation that incorporates an immune activating adjuvant. This will hold true for the design of a successful therapeutic HIV vaccine targeted at controlling reactivated virus following cessation of combined antiretroviral therapy (cART). The HIV accessory protein Nef functions by interfering with HIV antigen presentation through the major histocompatibility complex I (MHC-I) pathway thereby suppressing CD8^+^ cytotoxic T cell (CTL)-mediated killing of HIV infected cells. Thus, this important impediment to HIV vaccine success must be circumvented. This review covers our current knowledge of Nef inhibitors that may serve as immune adjuvants that will specifically restore and enhance CTL-mediated killing of reactivated HIV infected cells as part of an overall vaccine strategy to affect a cure for HIV infection.

## Background

Over the last 25 years, cART has evolved into a highly effective therapy that prolongs the lifespan of HIV infected individuals. However, it only targets actively replicating virus in permissive cells [1]. Indeed, infected individuals contain tissue reservoirs that replicate low levels of virus and may continually express low levels of Nef, one of HIV’s key pathogenicity-associated accessory proteins [2, 3]. The goals of current HIV cure research are to eliminate these reservoirs thereby purging infected patients of HIV. An integral portion of this strategy is the reactivation of latent virus (shock) and the subsequent elimination of the cells producing the reawakened HIV (kill) by the immune response. An IL-15 agonist, ALT-803, is currently in phase 2 trials due to its ability to enhance the CTL response [4]. However, shock and kill strategies will always be confronted with the ability of reactivated HIV-1 to produce a functional Nef protein that mediates critical HIV immune evasion effects. Indeed, HIV-1 Nef suppresses MHC-I antigen presentation in infected cells thereby blunting any therapeutic HIV vaccine efficacy.

## Nef alters host cellular trafficking pathways to attenuate the immune response

**Fig. 1:**
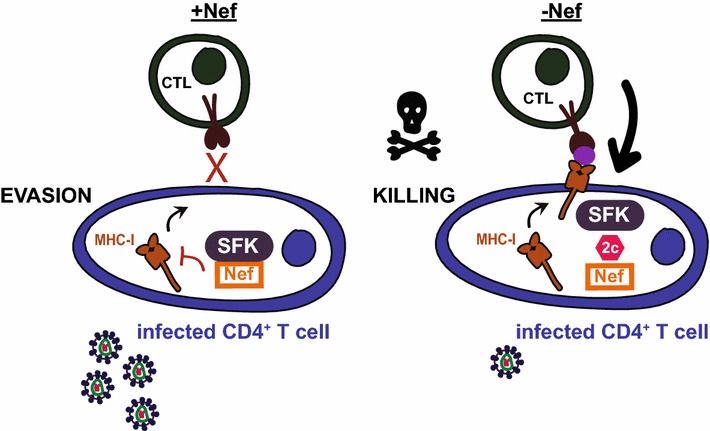
Shock and kill therapies to cure HIV infection require the inhibition of Nef activity. Shock therapies aim to reactivate latent HIV (shock and to eliminate virus-producing cells (kill). Viral reactivation will enhance Nef activity to evade the immune surveillance system by decreasing cell surface levels of MHC-I on CD4^+^ T cells (+Nef). Evasion: the interaction between Nef and a Src Family Kinase (SFK) results in the intracellular retention of MHC-I. Subsequently, a CD8^+^T lymphocyte (CTL) will fail to recognize an HIV infected cell. Killing: conversely, the inhibition of Nef’s activity using molecular adjuvants, such as 2c-like compounds (*red hexagon*) that block the interaction between Nef and SFKs (−Nef) will restore cell surface levels of MHC-I which in turn will promote HIV antigen presentation (*purple dot*) and enhance susceptibility to HIV specific CD8^+^-CTL

During the early stages of the HIV life cycle, the most heavily transcribed gene within infected cells is *nef*. This robust Nef expression affects infected cells in numerous ways, including downregulation of key cell surface receptors such as MHC-I and CD4 [3], enhancement of viral replication [5], alteration of T cell activation [6], and the subversion of the apoptotic machinery [7]. The downregulation of CD4 prevents superinfection of the cell and antibody-dependent cellular cytotoxicity [8, 9]. The downregulation of MHC-I attenuates the cytotoxic T-lymphocyte (CTL) recognition mechanism that seeks and destroys infected cells, allowing HIV-1-infected cells to evade the CTL immune response [10]. Nef’s ability to disrupt the CTL response is counter to the multiple shock and kill approaches currently used to target the latent reservoir of HIV-1 [1, 11] many of which include the use of a therapeutic vaccine prior to cessation of cART as part of a cure strategy. Thus, inhibition of Nef represents an essential arm of any anti-HIV shock and kill cure therapy that can restore and boost the efficacy of the anti-HIV CTL response (Fig. 1).

We have defined the mechanism used by Nef to downregulate cell surface MHC-I. This pathway, which ultimately mutes immune responses, requires the sequential use of multiple evolutionally conserved Nef motifs [3]. Indeed, conservation of these motifs in the pandemic M group of HIV-1, which is responsible for over 90% of AIDS cases worldwide, suggests they control essential pathways [12]. First, the Nef EEEE_65_ acidic cluster is required for trafficking Nef to paranuclear compartments, including an endosomal sub-population and the trans-Golgi network (TGN), through its interaction with the membrane trafficking regulator PACS-2 [13]. Second, the PxxP_75_ SH3 domain allows Nef to activate a TGN-localized Src Family Kinase (SFK) [14]. The activated Nef–SFK complex then recruits and activates the tyrosine kinase ZAP-70, which activates phosphatidylinositol-3 kinase (PI3K) triggering endocytosis of cell-surface MHC-I molecules which exhibit delayed recycling to the plasma membrane [15]. The membrane trafficking regulator PACS-1 would then contribute with the heterotetrameric adaptor protein-1 (AP-1) complex to sequester MHC-I away from the cell surface [13, 16, 17]. Thus, the Nef–SFK interaction attenuates the CTL response.

## The Nef PxxP_75_ site

The importance of the Nef–SFK interaction has been confirmed in vivo using a mouse model expressing Nef or a mutated Nef PxxP_75_ deficient in SFK binding (Nef AxxA_75_) in CD4^+^ T-cells, macrophages, and dendritic cells from the CD4 gene promoter (CD4C/HIV) [18]. Transgenic mice expressing Nef AxxA_75_ were completely protected from the AIDS-like phenotype induced by wild-type Nef, indicating an intact PxxP_75_ domain is critical for Nef-induced pathogenesis [18]. Thus, the Nef AxxA_75_ transgenic mouse model suggested that disruption of Nef-PxxP_75_ interactions (such as Nef–SFK) largely abolishes the pathogenic potential of Nef and prevents the AIDS-like phenotype.

Along with the Nef–SFK requirement in MHC-I downregulation, the inverse ability of Nef to enhance viral replication is also dependent on Nef–SFK interactions. Cells expressing Nef AxxA_75_ were deficient in Nef-mediated enhancement of viral replication by fivefold [19]. In addition, we described how disrupting the Nef–SFK interaction with the small molecule 2c blocked Nef-mediated MHC-I downregulation in human primary CD4^+^ T-cells [13]. Subsequently, 2c was also reported to inhibit Nef PxxP_75_-dependent HIV-1 infectivity and replication in macrophages [19]. Similar results were obtained in human monocytes using diphenylfuropyriminde compounds, which block Nef PxxP_75_-dependent HIV replication by directly inhibiting SFK activity [20]. Thus, small molecule inhibitors of the Nef PxxP_75_ interaction can repress two key aspects of Nef activity by: (1) preventing the downregulation of MHC-I which is key to disrupting the CTL response and (2) blocking Nef’s ability to enhance HIV replication in the primary human cells HIV normally replicates in and can exist latently in. Both are key elements required of any shock and kill therapeutic vaccine strategy to be an effective long-term cure for HIV infection strategy. Eliminating HIV infected cells while minimizing infection of new T cells will enhance the possibilities of eliminating HIV reservoirs.

## Conclusions

Due to Nef’s primary role in the pathogenesis of HIV-1, the identification of novel small molecules that block key aspects of Nef function creates an important opportunity for the generation of a new class of molecular adjuvants that will augment current therapeutic vaccine approaches such that maximal anti-HIV CTL activity can be achieved. Current efforts to generate and test a second generation of 2c-like Nef inhibitors with greater affinity for the Nef–SFK interface are under way. While 2c-like inhibitors block the Nef–SFK-mediated effects on MHC I surface expression and virus production, inhibitors that block the other functions of Nef still need to be found. Ultimately, this will lead the way to future non-human primate pre-clinical trials and subsequently to human trials. We believe a successful therapeutic HIV vaccine strategy applied pre-cART cessation necessitates the concomitant inhibition of Nef’s activity in order to maximize efficacy of shock and kill therapeutic strategies. Immunotherapies based on the MHC-I independent chimeric antigen receptor (CAR)-engineered T cells targeted specifically to HIV infected cells [21] will still benefit from the presence of 2c-like Nef inhibitors as they will ensure that virus production from reactivated T cells is kept to a minimum. In this way, vaccine- and CAR-based immunotherapies can be optimized for HIV-1 infected individuals such that the chances of a permanent cure may be realized.
